# On the Need for Better Exposure Assessment for Air Pollution with High Spatial and Temporal Variation

**DOI:** 10.3390/ijerph16091594

**Published:** 2019-05-07

**Authors:** Doug Brugge

**Affiliations:** 1 Department of Community Medicine and Health Care, University of Connecticut School of Medicine, 263 Farmington Ave., Farmington, CT 06030, USA; brugge@uchc.edu; 2 Department of Public Health and Community Medicine, Tufts University School of Medicine, 136 Harrison Avenue, Boston, MA 02111, USA

The mainstay of air pollution health research has been fine particulate matter pollution (PM_2.5_; aerodynamic diameter < 2.5 microns). PM_2.5_ is an appealing pollutant to study because its concentration tends to vary only slightly across relatively large geographic areas, such as a metropolitan region. Because of this, it is expected that there will be small exposure errors from assigning city-wide levels or interpolating between a few or several fixed site monitors spread over a city or region. Recently, satellite data has added another means to assess PM_2.5_ levels at resolutions of a square kilometer where there are few or no ground level monitors. These approaches have been quite successful at establishing PM_2.5_ as one of the top ten public health problems in the world [1].

However, other pollutants are not as well behaved as PM_2.5_. In particular, some pollutants, such as ultrafine particles (UFPs; <0.1 microns), course PM (2.5–10 microns), black carbon (BC), and oxides of nitrogen (which are gasses) vary substantially over short distances (tens of meters) and in time (hourly) [2,3]. Because these pollutants are elevated near highways and major roadways, there is substantial literature using proximity to these sources as surrogates for exposure [4].

However, there are other possible explanations for health effects based on proximity, including elevated traffic noise and lower socioeconomic status (SES) next to busy roadways [5]. In addition, proximity does not help with separating out the effects of different air pollutants in the complex mix created by motor vehicles. An important distinction, for example, is gasses vs. particles [6]. Thus, in order to inform policy in competent and accurate ways, there is a need for epidemiology studies that accurately assign exposure to locally elevated and highly variable pollutants from traffic.

The temporal and spatial variation of these pollutants is compounded by the mobility of people. Most individuals do not stay at home all day, so assigning their exposure based only on their home address will introduce error [7]. It is also potentially problematic to assume that ambient levels of pollution outside the home are the same as those indoors, where most people spend most of their time while at home [8]. Of course, this problem also applies to PM_2.5_, but the entry of pollutants has been shown to vary based on their own properties as well as building characteristics [9].

For the sake of illustration, let us consider two individuals living next to a freeway. One might be retired and spend most of their day indoors at home with brief trips out for errands midday when traffic is not as bad as it would be at rush hour. Their home might have no mechanical air handling system or air filtration. The other might work at a job 8 h per day at a distance that requires commuting on a highway (high exposure) to an office that is not near traffic and that has excellent ventilation systems that reduce exposure further. Ambient air pollution levels at the home might be reasonably good for the former and quite erroneous for the latter.

A daunting challenge for environmental exposure assessment and epidemiology is to find ways to reduce exposure error and improve confidence in health outcome associations for near traffic pollutants. The projects that I have participated in and helped lead under the umbrella of the Community Assessment of Freeway Exposure and Health (CAFEH: https://sites.tufts.edu/cafeh/) have focused on one of these pollutants, ultrafine particles or UFPs, in the Boston, MA, USA, area. I believe that our attempts to grapple with this problem, while not solving it, point to some important lessons.

The first lesson that I would point to is the need for ambient models of near highway pollutants that have fine grain temporal and spatial resolution. We have produced models using either mobile monitoring alone or with stationary fixed site monitoring to improve temporal prediction [10,11]. These models were at hourly resolution for a year with 20 m spatial resolution. We lost predictive power relative to models that produce only annual averages, but gained the ability to consider exposure based on time activity patterns of participants in our studies.

The second lesson is that address matching is insufficient because it can contain errors of up to 100 m, which is a large distance relative to the distances at which UFPs vary. One reason for this is that large multifamily developments can have a single address for apartments that extends over a block or more. In our first study, we resorted to positioning each address on an ortho map, including placing the participant within their apartment inside a multifamily building [7].

Third, we have monitored indoor-outdoor concentrations of UFPs in a subset of homes in our study as well as homes in a second (related) study [12,13]. The evidence from this work suggests that in the absence of forced air systems or free standing filters, UFPs infiltrate homes relatively well. This is likely because the homes in our study populations often have windows open and building envelopes that are relatively leaky even when windows are closed. Different populations, for example, people living in modern multifamily buildings with central ventilation, could have very different indoor concentrations compared to outdoor concentrations.

Fourth, adjusting exposure for participant time activity, as per the two hypothetical, but realistic, examples given above, changed assigned exposure for many participants. Of note, many near-highway residents were assigned lower exposures because their work took them away from the highway to less polluted areas for large parts of the day. We think these adjustments were an improvement in exposure assignment because exposure–response relationships became stronger and more monotonic after adjustment [14].

A final substantial factor to emerge from our analyses was that adjusting for confounding had an appreciable effect on the associations we observed. Body mass index was a leading (negative) confounder, as was race. We found that immigrants from East Asia, who were a large subpopulation in our sample, had much lower inflammatory responses to UFP exposure than the rest of our study population. Without making the adjustments just described, we would not have seen associations [15].

In addition to these primary lessons, we also explored some other factors that could affect exposure assignment and error. One was vertical dispersion of UFPs. We measured concentrations vertically from a tower near one of our neighborhoods and produced evidence that levels did not decline up to the fourth story or so and, accordingly, recruited only below this level [16]. We also used estimated respiratory rate to adjust exposure and have attempted to validate the calculated respiration rates with empirical data [17].

Despite our considerable attention to exposure error, we believe that substantial error remains. We are also unable, at this time, to extend these methods to large cohort studies. The reason for this is that the fine grain ambient models of UFP concentration do not transfer well from one geographic area to another [10]. Conducting the level of mobile and fixed site monitoring that we used for our models across large geographic regions, such as would be the case for most larger cohorts, would require unreasonable time and resources.

Most of the emerging UFP long-term exposure epidemiology uses annual averages for exposure [18]. This certainly has some advantages. For example, it has allowed other research teams to assign exposure to much larger populations than ours, potentially increasing statistical power. However, we suspect annual average exposures that are not adjusted for the factors we identified result in error that biases associations toward the null and contributes to small effect estimates that fall short of statistical significance.

The strategy of increasing power through larger sample sizes and of improving fine grain exposure assessment could, potentially, be complimentary. Presumably, as one increases sample size and loses exposure accuracy, one is gaining statistical power. Perhaps there is a “sweet spot” at which one could maximize the benefits of statistical power while minimizing exposure error using at least some of the techniques we have employed.

This is an area ripe for research innovation with the potential reward of informing policy development. UFPs were, for the first time this year, included as likely to be causally related to health (neurological) in the US Environmental Protection Agency’s Integrated Health Assessment for Particulate Matter [19]. Their inclusion, however, was based on controlled exposure animal and human toxicological studies, further emphasizing the need for definitive epidemiology studies to complement the work with animals.
